# Unveiling the hidden impact: metabolomic changes in children undergoing VSD repair

**DOI:** 10.1186/s12887-025-06083-9

**Published:** 2025-08-29

**Authors:** Yangyang Wu, Jiyi Huang, Yong Zou, Junkai Duan, Hongqiang Tu

**Affiliations:** 1 Cardiology Treatment Center, Jiangxi Provincial Children’s Hospital, 1666 Diezihu Road, Honggutan District, Nanchang, 330104 Jiangxi China; 2 JXHC Key Laboratory of Children’s Cardiovascular Diseases, Jiangxi Provincial Children’s Hospital, Nanchang, 330104 Jiangxi China; 3 Jiangxi Medical College, Nanchang University, Nanchang, 330006 Jiangxi China

**Keywords:** Ventricular septal defect, Cardiopulmonary bypass, Metabolomics, Molecular docking

## Abstract

**Background:**

We analyzed the perioperative metabolomic alterations in children undergoing ventricular septal defect (VSD) repair under cardiopulmonary bypass (CPB), identified evidence of postoperative injury, and explored strategies to mitigate such injuries.

**Methods:**

We conducted an untargeted metabolomic analysis of serum at three distinct time points (preoperative (Tp), immediate postoperative (T0), and 24 h postoperative (T24)) in eight children undergoing VSD repair under CPB. Subsequently, we identified the key enzymes associated with perioperative injury for molecular docking prediction studies.

**Results:**

We identified 623 metabolites in serum samples with VIP scores exceeding 1 in all three groups; 37 of these metabolites exhibited significant differences throughout the study phases. Three metabolic pathways—glycerophospholipid metabolism, arginine and proline metabolism, and retrograde endogenous cannabinoid signaling recurred in various comparisons between the two groups. Molecular docking predictions confirmed that arginine-glycine amidinotransferase may possess binding sites for bosentan and AdipoRon.

**Conclusion:**

The perioperative metabolic profiles in children undergoing VSD repair under CPB were significantly altered, presumably because of the inflammatory response and endothelial cell dysfunction induced by CPB. Molecular docking predictions suggested that bosentan and AdipoRon may be potent compounds that influence perioperative damage.

## Introduction

Ventricular septal defect (VSD) stands out as the most prevalent congenital heart disease, constituting approximately 40% of all cardiac anomalies [1]. Complications arising from VSD include pulmonary hypertension, Eisenmenger syndrome, and heart failure [2]. The widely adopted surgical approach for VSD repair involves direct visualization with cardiopulmonary bypass (CPB) through a chest incision. Although CPB provides surgeons with adequate visual field exposure, it induces a severe systemic inflammatory cascade and endothelial cell dysfunction [3]. Despite these well-acknowledged challenges, there has been a lack of early intervention strategies in clinical practice to address postoperative pathophysiological changes following CPB. Treatment protocols are typically instituted only after the manifestation of postoperative complications. Therefore, gaining a comprehensive understanding of the pathways underlying pathophysiological changes in children undergoing CPB is imperative. Such knowledge will aid in predicting and targeting interventions during the perioperative period, ultimately improving the prognosis for these children.

Metabolomics, the analysis of small-molecule metabolites in body fluids, cells, and tissues, facilitates the detection of subtle changes in biological pathways. It serves as a powerful tool for biomarker discovery, shedding light on disease pathogenesis [4]. In a preliminary untargeted study, Davidson et al. [5] observed significant changes in the postoperative metabolic profiles of children undergoing CPB by analyzing targeted metabolites in their serum. Aspartate, glutamate, methyl nicotinamide, trigonelline, and kynurenine emerged as potential differentiators between critically ill patients after CPB and those with a less severe disease course. Wu et al. [6] conducted an experimental animal study using tissues from various organs of fetal sheep undergoing CPB. Their untargeted metabolomic analysis revealed substantial metabolic changes, particularly altered amino acid metabolism, dysregulation of lipid metabolism, and accumulation of plasticizer metabolites. This study also highlighted significant alterations in cardiac function. In summary, metabolic profile changes can serve as indicators of injury throughout the perioperative period of CPB, warranting further investigation to identify novel therapeutic targets.

This study aimed to conduct an untargeted metabolomic analysis of the serum of children undergoing surgical repair with CPB and to map the metabolic profile to identify alterations before and after surgery. In addition, this study aimed to predict the perioperative period and implement targeted interventions to enhance the outcomes of these children.

## Materials and methods

### Participants

The study included pediatric patients diagnosed with VSD who underwent repair under CPB at Jiangxi Provincial Children’s Hospital between April 12 and May 12, 2023. In addition, all of the children had no special dietary or pharmacological treatment during the perioperative period of the study period. Exclusion criteria were defined as follows: (1) broken clinical data, (2) presence of organic cardiac changes other than patent foramen ovale, (3) concomitant organ or systemic diseases, (4) concomitant endocrine-genetic metabolic disorders, (5) concomitant hereditary disorders, and (6) previous surgery. Informed consent was obtained from all parents or guardians of participating children. The consent process followed the guidelines of the Medical Ethics Committee of Jiangxi Provincial Children’s Hospital (approval Code: JXSETYY-YXKY-20230086).

### Sample collection

Serum samples were obtained from all patients at three distinct time points: preoperatively, immediately postoperatively, and 24 h postoperatively. Within 2 h of serum collection, the samples were subjected to centrifugation at 4 °C. The separated serum was then dispensed into RNAase-depleted cryopreservation tubes and promptly submerged in liquid nitrogen at −196 °C for a minimum of 15 min. Subsequently, the samples were shifted to a refrigerator at −80 °C for long-term storage. Concurrently, pertinent clinical data were gathered from the patients both before and 24 h after surgery.

### Metabolomics analysis

The analysis was conducted using an ultra-high performance liquid chromatography (UHPLC) system (1290 Infinity LC, Agilent Technologies) coupled with a quadrupole time-of-flight mass spectrometer (AB Sciex TripleTOF 6600) from Shanghai Applied Protein Technology Co., Ltd.

Serum samples were analyzed by hydrophilic interaction liquid chromatography (HILIC) separation using a 2.1 mm × 100 mm ACQUIY UPLC BEH 1.7 μm column (Waters, Ireland). The mobile phase consisted of 25 mM ammonium acetate and 25 mM ammonium hydroxide in water for component A and acetonitrile for component B in both electrospray ionization (ESI) positive and negative modes. The gradient elution profile was as follows: starting with 85% B for 1 min, linear decreasing to 65% over 11 min, further decreasing to 40% in 0.1 min with a 4 min hold, increasing to 85% B over 0.1 min, ending with 5 min of re-equilibration.

The RPLC separation was carried out on a 2.1 mm × 100 mm ACQUIY UPLC HSS T3 1.8 μm column (Waters, Ireland). In ESI-positive mode, the mobile phase consisted of water containing 0.1% formic acid as component A and acetonitrile containing 0.1% formic acid as component B. Conversely, in ESI-negative mode, the mobile phase comprised of A = 0.5mM ammonium fluoride in water and B = acetonitrile. The gradient elution profile was as follows: starting at 1%B for 1.5 min, the gradient increased linearly to 99% within 11.5 min and was held for 3.5 min. The gradient then decreased to 1% in 0.1 min, followed by a re-equilibration period of 3.4 min. The gradient flow rate was 0.3 mL/min and the column temperature was kept constantly at 25 °C. Aliquots of 2 µL of each sample were injected.

Following the HILIC chromatographic separation, the ESI source obtained the following conditions: Ion Source Gas1 (Gas1) at 60, Ion Source Gas2 (Gas2) at 60, curtain gas (CUR) at 30, source temperature at 600 ℃, and IonSpray Voltage Floating (ISVF) at ± 5500 V; TOF MS scan m/z range: 60-1000 Da, product ion scan m/z range: 25-1000 Da, TOF MS scan accumulation time 0.20 s/spectra, product ion scan accumulation time 0.05 s/specta. The secondary mass spectra were performed using information dependent acquisition (IDA) and in high sensitivity mode, collision energy (CE) at 35 V with ± 15 eV; declustering potential (DP) at 60 V (+) and − 60 V (−); IDA settings were as follows: exclude isotopes within 4 Da, and dandidate ions to monitor per cycle at 10.

With Compound Discoverer 3.2 software (Thermo Fisher Scientific, Waltham, MA, USA), raw liquid chromatography-mass spectrometry (LC-MS) data were processed to identify statistically significant (*P*-value < 0.05, fold change [FC] > 1) ion peaks, and compare the MS information with databases including Human Metabolome, ChemSpider, and mzCloud. Multivariate statistical analysis and data visualization were performed by R software, involving principal component analysis (PCA), partial least squares discriminant analysis (PLS-DA), orthogonal partial least squares discriminant analysis (OPLS-DA), Venn diagrams, volcanic map, and heat maps. The threshold for differences was set at VIP ≥ 1 and *t*-test *P* < 0.05 in the OPLS-DA model. Whereas, significantly differentially expressed metabolites were enriched for KEGG with the use of MetaboAnalyst 5.0 (https://www.metaboanalyst.ca/).

### Molecular docking

The molecular affinity between the small-molecule compounds and proteins was validated through molecular docking. The protein crystal structures were sourced from the RCSB Protein Data Bank (PDB) (https://www.rcsb.org/), whereas the 3D molecular structures of the compounds were retrieved from the ZINC database (https://zinc.docking.org/). Molecular docking simulations were conducted using AutoDock [7], and only the target proteins documented with ligands in the PDB were chosen for analysis. To ensure the reliability of the docking model, a redocking process was used for validation.

### Statistical analysis

The results are all presented as mean ± standard deviation (SD). Data analysis and graphics are produced with the R toolkit. Furthermore, the patients’ clinical and metabolomic data were statistically analyzed for differences between the two groups using paired *t*-tests, and multiple comparisons were made using one-way analysis of variance (ANOVA). *P* < 0.05 was accepted as statistically significant.

## Results

### Analysis of clinical data

After identifying the study participants and collecting their clinical data, we analyzed the data (Table 1) and observed a significant decrease in erythrocyte counts, hemoglobin content, and erythrocyte ratio in blood counts. This finding may indicate the destruction and loss of blood cells due to CPB. Concurrently, the data revealed a notable increase in inflammatory markers, including C-reactive protein level, neutrophil percentage, white blood cell count, and procalcitonin level. These results confirm a severe systemic inflammatory response to CPB. Substantial postoperative elevations in urea and indicators for evaluating myocardial function were also observed. These effects are consistent with the adverse outcomes associated with CPB.

**Table 1:** Comparison of preoperative and postoperative clinical indicators in children receiving CPB^a^

Pairs		Mean	Std. Deviation	Std. Error Mean	95% Confidence Interval of the Difference		t	df	Sig. (2-tailed)
					Lower	Upper			
Pair 1	RBC - RBC’	0.91000	0.55621	0.19665	0.44500	1.37500	4.627	7	0.002
Pair 2	WBC - WBC’	−5.62500	2.86000	1.01116	−8.01602	−3.23398	−5.563	7	0.001
Pair 3	PLT - PLT’	77.75000	73.25640	25.90005	16.50612	138.99388	3.002	7	0.020
Pair 4	Hb - Hb’	20.62500	16.52649	5.84300	6.80851	34.44149	3.530	7	0.010
Pair 5	N - N’	−41.73750	19.76425	6.98772	−58.26083	−25.21417	−5.973	7	0.001
Pair 6	L - L’	39.41250	17.47214	6.17733	24.80543	54.01957	6.380	7	0.000
Pair 7	M - M’	−1.06750	3.86832	1.36766	−4.30149	2.16649	− 0.781	7	0.461
Pair 8	PCV - PCV’	7.15000	5.14087	1.81757	2.85212	11.44788	3.934	7	0.006
Pair 9	PCT - PCT’	0.06875	0.07511	0.02655	0.00596	0.13154	2.589	7	0.036
Pair 10	TBIL - TBIL’	−4.30000	8.51201	3.00945	−11.41622	2.81622	−1.429	7	0.196
Pair 11	DBIL - DBIL’	− 0.68000	1.26341	0.44668	−1.73624	0.37624	−1.522	7	0.172
Pair 12	IBIL - IBIL’	−3.62000	7.31212	2.58522	−9.73308	2.49308	−1.400	7	0.204
Pair 13	LDH - LDH’	−182.75000	123.04255	43.50211	−285.61614	−79.88386	−4.201	7	0.004
Pair 14	UR - UR’	−2.48500	2.13224	0.75386	−4.26760	− 0.70240	−3.296	7	0.013
Pair 15	CR - CR’	1.65000	9.79344	3.46250	−6.53752	9.83752	0.477	7	0.648
Pair 16	Tn1 - Tn1’	− 0.01750	0.18053	0.06383	− 0.16843	0.13343	− 0.274	7	0.792
Pair 17	CK - CK’	−1015.31250	591.75462	209.21685	−1510.03175	−520.59325	−4.853	7	0.002
Pair 18	CK-MB - CK-MB’	−12.70000	13.01735	4.60233	−23.58278	−1.81722	−2.759	7	0.028
Pair 19	NT-proBNP - NT-proBNP’	−2294.50000	3019.00784	1509.50392	−7098.41517	2509.41517	−1.520	3	0.226
Pair 21	CRP - CRP’	−23.60125	8.89018	3.14315	−31.03363	−16.16887	−7.509	7	0.000

’indicates post-operative data

a. RBC: red blood cell count; WBC: white blood cell count; PLT: platelet count; Hb: hemoglobin content; N: percentage of neutrophils; L: percentage of lymphocytes; M: percentage of monocytes; PCV: specific volume of erythrocytes; PCT: platelet crit; TBIL: total bilirubin; DBIL: direct bilirubin; IBIL: indirect bilirubin; LDH: lactic acid dehydrogenase; UR: urea; ER: creatinine; Tn1: troponin-1; CK: creatine kinase; CK-MB: creatine kinase isoenzyme (activity); NT-proBNP: N-terminal B-type natriuretic peptide pro. All data were statistically analyzed using the *t*-test

### Modification of metabolite profiles in children undergoing surgical repair of VSD under CPB

**Fig. 1:**
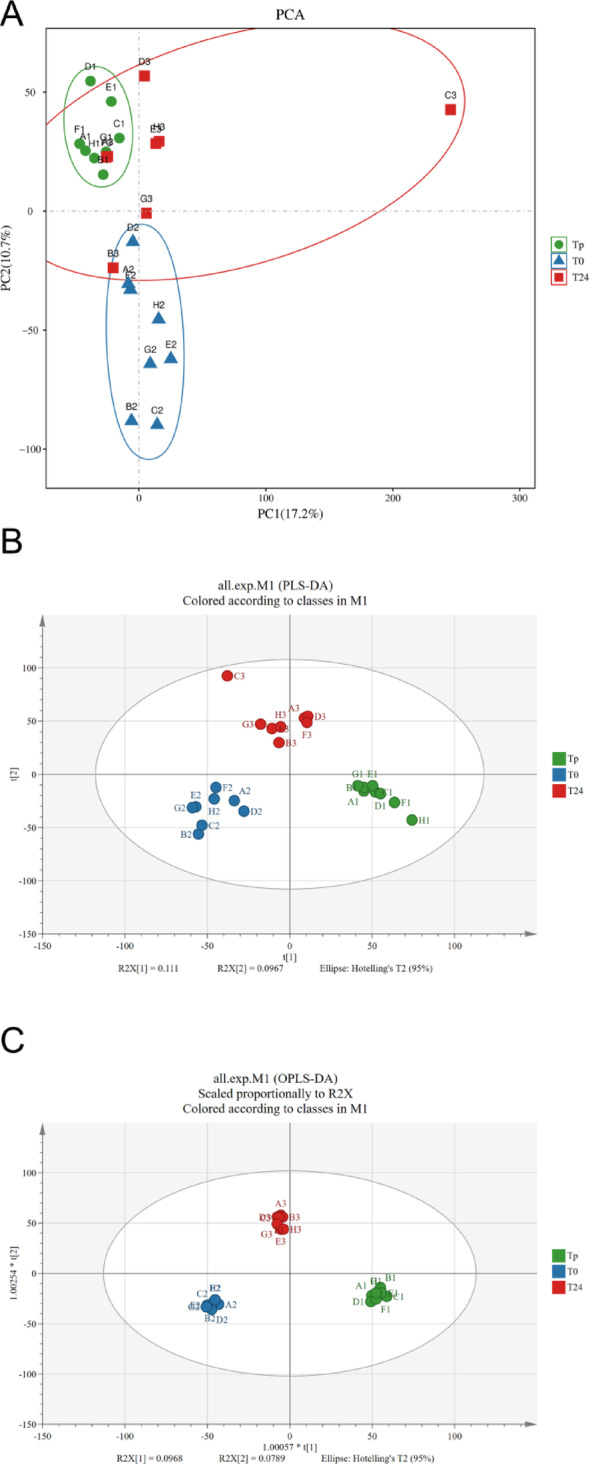
Results of PCA, PLS-DA, and OPLS-DA analysis. **A**: PCA analysis. **B**: PLS-DA analysis. **C**: OPLS-DA analysis

**Fig. 2:**
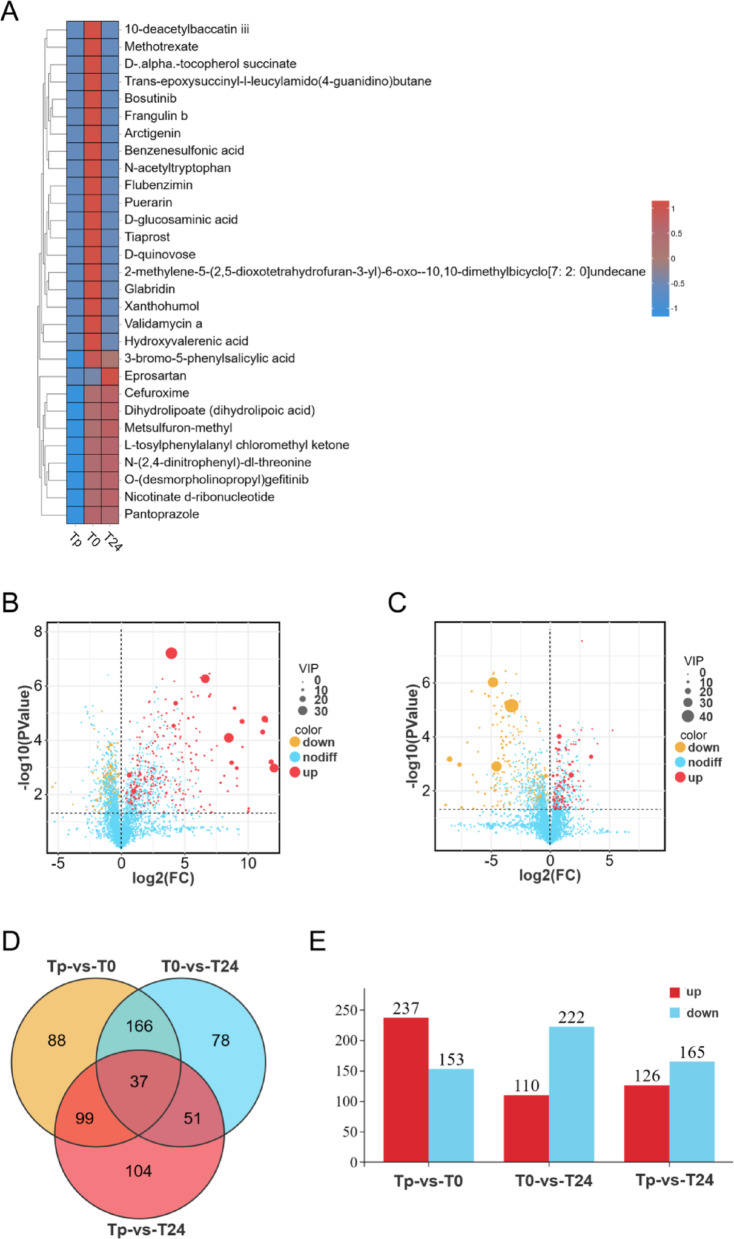
Results of postoperative metabolite differential analysis in children undergoing CPB. **A**: Heat map displaying 29 metabolites with a fold-change (FC) > 100 or < 0.01. **B**: Differential metabolite volcano plots for Tp-vs-T0. **C**: Differential metabolite volcano plots for T0-vs-T24. **D**: Wayne plots illustrate the number of significantly different metabolites among the three groups. **E**: Bar graph of significantly different metabolites among the three groups

Non-targeted metabolomics was employed to analyze the serum metabolites in eight children diagnosed with VSD. Serum samples were collected at three distinct time points (Tp, T0, and T24) during the perioperative period, followed by LC-MS analysis. Subsequently, we assessed the sample distribution at different time points using PCA, PLS-DA, and OPLS-DA. The results showed significant differences in the two-dimensional spatial distribution among all groups (Tp, T0, and T24), indicating the representative and biologically reproducible nature of the data (Fig. 1). It can be seen that the results of OPLS-DA are satisfactory. Therefore, we combined the VIP value of OPLS-DA and the p-value of t-test to perform the analysis of variance. The results showed that a total of 623 metabolites in the serum samples exhibited VIP scores > 1 across all three groups, with 37 metabolites showing significance throughout the study phases. Of these, 29 metabolites were depicted in the heatmap, demonstrating an FC greater than 100 or less than 0.01. At the same time, the differences between Tp-vs-T0 and T0-vs-T24 were further demonstrated by plotting their differential metabolites as volcano plots. In the comparison of Tp versus T0, 390 metabolites in the serum samples exhibited alterations (VIP > 1, *P* < 0.05), with 237 increased and 153 decreased. Comparing T0 and T24, significant differential changes were observed in serum samples involving 110 metabolite elevations and 222 metabolite reductions, respectively (VIP > 1, *P* < 0.05). (Fig. 2).

**Fig. 3:**
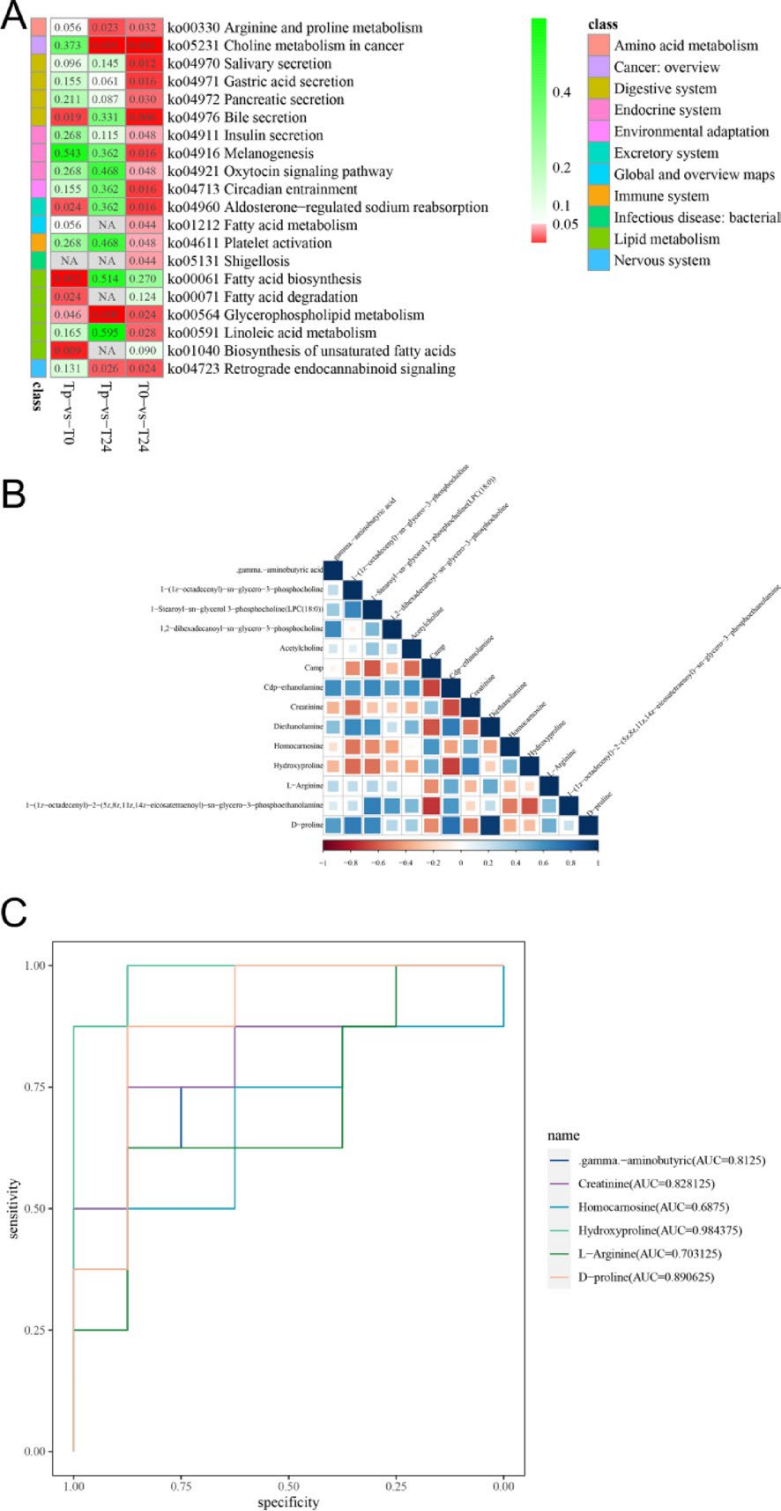
The results of KEGG pathway enrichment. **A**: The top 20 pathways analyzed for KEGG pathway enrichment. The numbers in the grid represent *P*-values, and the metabolic pathways are classified on the far left. **B**: Heatmap of differential metabolite correlations in three metabolic pathways in the Tp versus T0 comparison group. Blue represents a positive correlation and red a negative correlation, the greater the correlation the darker the colour. **C**: ROC curves of differential metabolites within the arginine and proline metabolic pathways in the Tp versus T0 comparison group

To assess the enrichment of significantly differentiated metabolites within specific pathways, we conducted the enrichment analysis on the metabolomic dataset using the web-based tool MetaboAnalyst (v5.0; https://www.metaboanalyst.ca). The analysis identified the top 20 enriched pathways (Fig. 3A). Tp exhibited higher concentrations of lipid metabolism, amino acid metabolism, and the digestive system than T0. Furthermore, lipid metabolism, amino acid metabolism, nervous system, and digestive system were more prominent in Tp than in T24. Three recurring metabolic pathways, namely glycerophospholipid metabolism, arginine and proline metabolism, and retrograde endogenous cannabinoid signaling, were identified in both groups. Subsequently, we extracted all differential metabolites from the three metabolic pathways in the Tp versus T0 comparison group for correlation analysis (Fig. 3B). At the same time, we utilized the differential metabolites in the arginine and proline metabolic pathways for receptor operating characteristic curves (ROC curves) (Fig. 3C). In addition, we investigated trends in differential metabolites among the three groups of serum samples. Interestingly, most metabolites that differed significantly in the glycerophospholipid metabolic pathway exhibited a downward trend over the study period. Conversely, most metabolites within the arginine and proline metabolic pathways exhibited an upward trend.

### Molecular Docking of the small molecule compounds Bosentan and adiporon with arginine-glycine amidinotransferase

**Fig. 4:**
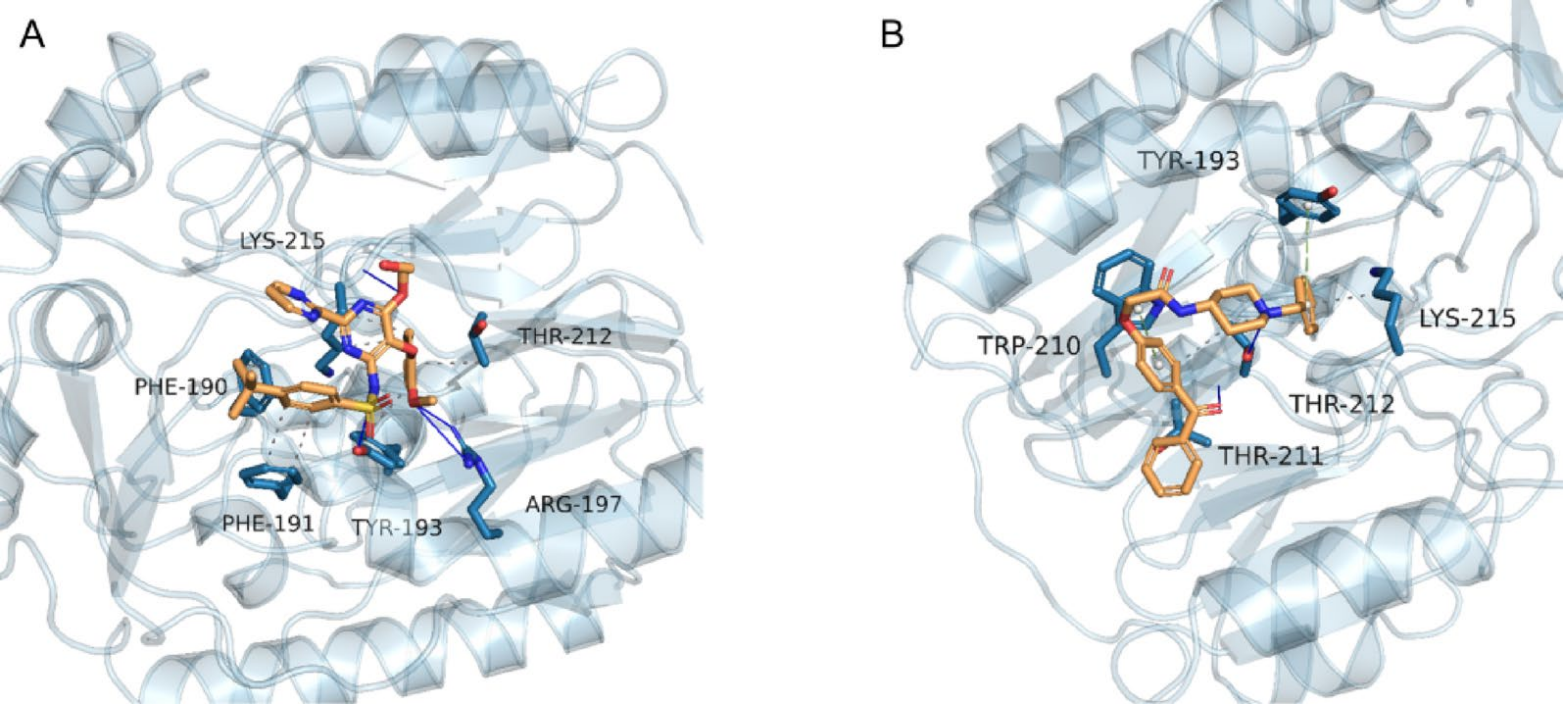
Molecular docking results. Orange represents ligand, blue represents receptor, white balls represent aromatic ring centers, grey dotted lines represent hydrophobic interactions, blue solid lines represent hydrogen bonding, and green dotted lines represent π-stacking (perpendicular). **A**: Bosentan and arginine-glycine amidinotransferase. **B**: AdipoRon and arginine-glycine amidinotransferase

To explore deeper into this investigation, we observed a significant elevation in creatinine levels and creatine by analyzing the differential metabolites within the arginine and proline metabolic pathways. Consequently, we propose that the arginine-glycine amidinotransferase in this pathway serves as a pivotal enzyme relevant to our study. We performed molecular docking studies involving bosentan, an endothelin receptor antagonist commonly used clinically to reduce pulmonary hypertension, and AdipoRon [8], a lipocalin receptor 1 and 2 agonist identified through a PEBMED database search as potentially linked to the modulation of CPB-induced inflammation and cardiac dysfunction. These studies were conducted with arginine-glycine amidinotransferase using AutoDock software to predict the presence of a binding site. Molecular docking results, wherein lower docking energies indicate greater binding capacity, demonstrated potential binding sites for both bosentan and AdipoRon with arginine-glycine amidinotransferase (Fig. 4), with energies below − 5.0 kcal/mol suggesting the possibility of binding. Consequently, our findings indicate that bosentan and AdipoRon have the potential to bind to arginine-glycine amidinotransferase, influencing post-CPB injury changes.

## Discussion

To the best of our knowledge, there has been a limited exploration of metabolomic analyses in children undergoing surgical repair of VSD under CPB. In a study by Davidson et al. [5], the perioperative metabolism of 165 children undergoing cardiac surgery was analyzed. The most significant changes in amino acid metabolism, such as arginine and proline metabolism, were found in children during the perioperative period of CPB. This finding follows the results of our study. Of these, aspartate, glutamate, nicotinamide methyl ester, tritiated alkaloids, and kynurenine were identified as useful in determining the postoperative prognosis of the children. Meanwhile, Wu et al. [6] observed significant changes in both lipid metabolism and amino acid metabolism by studying metabolic changes in fetal sheep undergoing CPB, a finding that is also mentioned in our study.

Our study represents the first comprehensive examination of metabolomic changes in serum samples from children with VSD during the perioperative correction of defects under CPB using molecular docking techniques. In our patient analysis, we observed significant alterations in 623 metabolites at different time points (Tp, T0, and T24). Subsequent KEGG enrichment analysis revealed three recurrent metabolic pathways—glycerophospholipid metabolism, arginine and proline metabolism, and retrograde endogenous cannabinoid signaling—in various comparisons between the two groups. These findings suggest that the perioperative metabolic profile changes in children undergoing surgical repair of VSD under CPB predominantly involve lipid and amino acid metabolism. Alterations in lipid metabolism concurrently affect the digestive, immune, and nervous systems. These changes within metabolic pathways may play a crucial role in the perioperative pathophysiological processes of children undergoing surgical repair of VSD under CPB.

Glycerophospholipids (GPL) are crucial components of cell membranes and their metabolism produces various bioactive lipid molecules, including inositol triphosphate, diacylglycerol, arachidonic acid, phosphatidic acid, and lysophosphatidic acid. These molecules regulate diverse signaling pathways within cells [9]. Substantial evidence supports the notion that glycerophospholipid metabolism plays a pivotal role in systemic immunity and low-grade inflammatory states, thereby implicating GPL as a potential mediator of inflammation [10, 11].

At the systemic level, the metabolism of arginine and proline ultimately yields various biochemically diverse products including proline, glutamate, creatine, urea, polyamines, nitric oxide, etc. Arginine plays a pivotal role in the urea cycle and has immunomodulatory and anti-inflammatory properties [10]. Other members of the urea cycle, such as ornithine and citrulline, are negatively associated with inflammation [11]. In contrast, arginine serves as a precursor of NO synthesis. NO functions as a crucial modulator of vascular endothelial dysfunction by influencing vasodilation and platelet aggregation and inhibiting smooth muscle proliferation [12]. While our findings suggest a potential defect in NO production through L-arginine, reduced NO production in endothelial cells and elevated levels of reactive oxygen species contribute to the decreased resistance of endothelial cells to neutrophil-induced cytotoxicity [13].

The endogenous cannabinoid system (ECS) is a lipid signaling system comprising endogenous cannabinoid-like ligands derived from arachidonic acid, including anandamide (AEA) and 2-arachidonoylglycerol (2-AG) [14]. The enrichment of retrograde endogenous cannabinoid signaling observed in our study may be attributable to alterations in perioperative lipid metabolism in children undergoing surgical repair of VSD under CPB. Alternatively, perioperative fasting can potentially modulate retrograde endogenous cannabinoid signaling.

Following our metabolomic analysis, we identified an arginine-glycine amidinotransferase from the arginine and proline metabolic pathways as a crucial focus for further investigation. In children undergoing surgical repair of VSD under CPB, bosentan and AdipoRon were found to modulate perioperative metabolic changes by binding to arginine-glycine amidinotransferase, thereby influencing perioperative injury.

Endothelin-1 (ET-1) is a potent vasoconstrictor that plays key roles in mediating cell proliferation, fibrosis, and inflammation [15]. Activation of endothelin receptor A induces pulmonary vasoconstriction and smooth muscle cell proliferation, whereas endothelin receptor B eliminates ET-1, mediating endothelial cell vasodilation, and release of prostacyclin and nitric oxide. Bosentan, an endothelin receptor antagonist, competitively inhibits ET-1 expression, and reduces pulmonary vascular resistance [16]. Previous studies in rats demonstrated that bosentan lowers blood pressure, prevents cardiac hypertrophy, and exhibits anti-inflammatory and antithrombotic effects [17]. Our identification of the binding site of bosentan on arginine-glycine amidinotransferase provides valuable insights into its potential mechanism of action.

Lipocalin is secreted by adipose tissue and binds to the lipocalin receptor, exerting its antidiabetic effects in the liver and skeletal muscle by activating the AMP-activated protein kinase (AMPK) and peroxisome proliferator-activated receptor (PPAR) alpha pathways. It ameliorates vascular dysfunction by activating endothelial NO production and exhibits anti-atherosclerotic effects on the vasculature by inhibiting various inflammatory conditions [18, 19]. It was shown in a recent animal study by Jenke et al. [8] that AdipoRon, a lipocalin receptor agonist, can attenuate CPB-induced cardiac impairment and inflammation. This attenuation occurred through AMPK-mediated inhibition of proinflammatory TLR4 and TNF-α signaling. In addition, the immunosuppressive IL-10 is upregulated in cardiac cells. These findings are consistent with those of the present study.

However, this study is subject to certain limitations. First, the sample size was restricted to eight patients, warranting the inclusion of a larger cohort for metabolomic analysis to enhance the robustness of the study. Second, the absence of fundamental experiments to assess the activity and expression of key enzymes within the metabolic pathway may have influenced the reliability of our findings. Meanwhile, to control the variables we selected children with VSD for the study, so the generalisability of the results needs to be further explored. In addition, it is crucial to acknowledge that the molecular docking results hold only a predictive value, necessitating supplementary experiments for further validation. However, drug applicability and potential adverse effects require further investigation.

## Conclusion

Significant differences were observed in the perioperative metabolic profiles of children undergoing surgical repair of VSD under CPB. Moreover, based on the distinct metabolite enrichments, three metabolic pathways—glycerophospholipid metabolism, arginine and proline metabolism, and retrograde endogenous cannabinoid signaling—recurred in both groups. These pathways may be associated with perioperative injuries in children undergoing CPB. Bosentan and AdipoRon have been identified as potential modulators of perioperative metabolic alterations in children with surgically repaired VSD under CPB, acting by binding to arginine-glycine amidinotransferase, a key enzyme in the arginine and proline metabolic pathways. Despite the limitations of our study, we plan to conduct further research to mitigate perioperative injuries in patients undergoing CPB.

## Data Availability

The datasets used and/or analysed during the current study are available from the corresponding author on reasonable request.
